# High levels of genetic differentiation and selfing in the Brazilian cerrado fruit tree *Dipteryx alata* Vog. (Fabaceae)

**DOI:** 10.1590/S1415-47572010005000007

**Published:** 2010-03-01

**Authors:** Roberto Tarazi, Maria Andréia Moreno, Flávio Bertin Gandara, Elza Martins Ferraz, Mário Luiz Teixeira Moraes, Christina Cleo Vinson, Ana Yamaguishi Ciampi, Roland Vencovsky, Paulo Yoshio Kageyama

**Affiliations:** 1 Laboratório de Reprodução e Genética de Espécies Arbóreas, Departamento de Ciências Florestais, Escola Superior de Agricultura Luiz de Queiroz, Universidade de São Paulo, Piracicaba, SPBrazil; 2 Departamento de Genética, Escola Superior de Agricultura Luiz de Queiroz, Universidade de São Paulo, Piracicaba, SPBrazil; 3 Departamento de Fitotecnia, Tecnologia de Alimentos e Sócio Economia, Universidade Estadual Paulista Júlio de Mesquita Filho', Ilha Solteira, SPBrazil; 4 Laboratório de Genética Vegetal, Embrapa Recursos Genéticos e Biotecnologia, Empresa Brasileira de Pesquisa Agropecuária, Brasília, GOBrazil

**Keywords:** *Dipteryx alata*, genetic structure, mating system, SSR

## Abstract

*Dipteryx alata* is a native fruit tree species of the cerrado (Brazilian savanna) that has great economic potential because of its multiple uses. Knowledge of how the genetic variability of this species is organized within and among populations would be useful for genetic conservation and breeding programs. We used nine simple sequence repeat (SSR) primers developed for *Dipteryx odorata* to evaluate the genetic structure of three populations of *D. alata* located in central Brazil based on a leaf sample analysis from 101 adults. The outcrossing rate was evaluated using 300 open-pollinated offspring from 25 seed-trees. Pollen dispersal was measured by parentage analysis. We used spatial genetic structure (SGS) to test the minimal distance for harvesting seeds in conservation and breeding programs. Our data indicate that the populations studied had a high degree of genetic diversity 
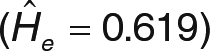
 and population structure, as suggested by the high level of divergence among populations 
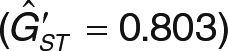
. The estimated outcrossing rate 
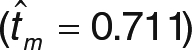
 suggested a mixed mating system, and the intrapopulation fixation index was influenced by SGS. We conclude that seed harvesting for genetic conservation and breeding programs requires a minimum distance between trees of 196 m to avoid collecting seeds from related seed-trees.

## Introduction

The cerrado (Brazilian savanna) is the second largest Brazilian biome and a hotspot of global biodiversity (Myers *et al.*, 2000). This hotspot designation was based on the biological diversity of the region, the number of endemic plant species and the urgent need to protect this region from major human impacts (Myers *et al.*, 2000). The intense and disorderly exploitation by agricultural expansion and livestock production has modified more than 50% of the cerrado (Klink and Machado, 2005). Large populations of notable native food resources such as *Dipteryx alata* and *Caryocar brasiliense* have been destroyed and continue to be neglected (Pott and Pott, 2003). Currently, with the need for conservation, forest restoration and food security there is a great demand for genetic information on native plants.

Among cerrado plants with a potential for exploitation, *D. alata*, locally known as “baru”, has a wide range of applications, *e.g.*, as human and animal food, a substrate for the pharmaceutical industry and construction wood (Siqueira *et al.*, 1993). This species is a native fruit tree of the family Fabaceae, widely distributed in the cerrado (Ribeiro and Walter, 2000). The hermaphroditic flowers of *D. alata* are pollinated by diverse insects and its seeds are gravity- and animal-dispersed (Ribeiro and Walter, 2000). The genetic variability among populations of *D. alata* was described by Soares *et al.* (2007) using random amplified polymorphic DNA (RAPD). Because of the dominant nature of RAPD markers, the levels of within-population inbreeding were estimated using restricted models and the mating system was not determined, thus leaving an information gap that needs to be filled.

Microsatellite markers, or simple sequence repeats (SSR), have been used in natural population studies as they are codominant and highly polymorphic when compared with other classes of markers. SSR markers have been widely used to address several questions in conservation and population genetics, such as population structure, intra-population spatial genetic structure, contemporary pollen and seed dispersal and mating systems (Bittencourt and Sebbenn, 2007; Dick *et al.*, 2008; Hanson *et al.*, 2008).

The main objective of this study was to obtain genetic information that could be useful for the domestication and breeding of *D. alata*, and its conservation. The genetic diversity, population structure, gene flow and mating system of this species were studied using nuclear SSR primer pairs originally developed for *Dipteryx odorata.*

## Material and Methods

###  Genetic material

**Figure 1:**
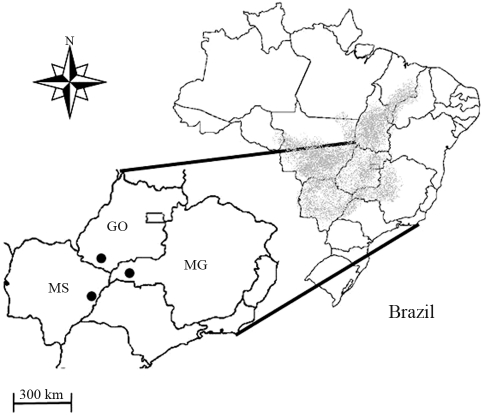
*Dipteryx alata* geographic distribution in the Brazilian cerrado biome (in gray) and the locations of the populations that were studied (•).

Leaf samples from 101 adult trees of *D. alata* were collected from three populations (Figure 1) at least 219 km apart in the State of Minas Gerais (MG), municipality of Campina Verde (n = 30; 19°31'35” S, 50°01'23” W), in the State of Goias (GO), municipality of Itarumã (n = 30; 18°44'36” S, 51°14'58” W), and in the State of Mato Grosso do Sul (MS), municipality of Brasilândia (n = 41; 21°14'24” S, 52°01'12” W). All trees in the populations were located in pastures at a low population density, where they had been left after land clearing for agricultural expansion with the sole purpose of offering shade for livestock. The geographic location of the adults in all of the areas studied was mapped with a global positioning system (GPS) and leaf samples for genetic analysis within a population were collected from trees separated by an average distance of 40 m. The mating system was studied using open-pollinated seeds collected from 25 seed-trees in the MS population. Twelve seedlings were randomly sampled from each seed-tree, *i.e.*, a total of 300 offspring.

###  Microsatellite markers and DNA isolation

DNA was extracted from the leaves of adult trees and offspring by using the CTAB method described by Doyle and Doyle (1987). DNA was quantified by agarose gel electrophoresis and diluted to a concentration of 2.5 ng/ μL. Each amplification reaction (12.74 μL) contained: 3 μL of genomic DNA, 1.3 μL of 10X PCR buffer (10 mM of Tris-HCl, 50 mM of KCl, 1,5 mM of MgCl_2_, pH 8.3), 4.30 μL of forward and reverse primers (1 μM), 0.2 μL of *Taq* DNA polymerase (5 U/ μL, Phoneutria); 1.3 μL of BSA (2.5 μg/ mL) and 1.34 μL of ultrapure water. Amplifications were done using an MJ Research PT-100 thermal cycler adjusted to the following conditions: 5 min at 94 °C, 30 cycles of 1 min at 95 °C, 1 min at the specific temperature for each primer (Table 1), 1 min at 72 °C, and a final elongation step of 7 min at 72 °C. The amplification products were separated on 5% (w/ v) polyacrylamide gels by electrophoresis at 55 W for 1.5 h. A 10 bp ladder (Invitrogen®) was used as a size marker. The gels were stained with silver nitrate (Creste *et al.*, 2001). Since microsatellite primers have not yet been developed for *D. alata*, we used 42 primer pairs previously developed by one of us for *D. odorata* (Vinson CC, MSc dissertation, Universidade Federal do Pará, Belém, 2004), obtained from CENARGEN-Embrapa (National Research Center of Genetic Resources and Biotechnology – Brazilian Agricultural Enterprise).

###  Genetic diversity analysis

The mean number of alleles per locus (*A*), the mean observed (*H*_*o*_) and expected (*H*_*e*_) heterozygosities, and the fixation indices for each population (*f*_*i*_) were estimated by using the program FSTAT 2.9.3 (Goudet, 2002). The significance of the *f*_*i*_ values was tested using a Monte-Carlo permutation approach implemented in FSTAT 2.9.3. This program was also used to test for deviation from Hardy-Weinberg equilibrium and linkage disequilibrium, with 1000 allelic permutations among individuals and a P-value corrected for a significance level of *a* = 0.05. Multiple tests were done with the Bonferroni correction.

Genetic structure was quantified using the Weir and Cockerham (1984) parameters (*f* = *F*_*IS*_, *F* = *F*_*IT*_ and θ_*p*_ = *F*_*ST*_). Since most microsatellite mutations involve the addition or subtraction of a small number of repeat units according to a stepwise mutation model, population differentiation was also assessed by Slatkin's corrected *R*_*ST*_ coefficient (Goodman, 1997). To improve the information about interpopulation genetic differentiation, we also used the correction proposed by Hedrick (2005) for the *G*_*ST*_ measure (Nei, 1973), namely 
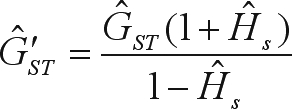
, where *H*_*s*_ is the intrapopulation genetic diversity (Nei, 1973). These statistics were computed with a significance test using FSTAT.

The historic gene flow (*Nm*) among populations was estimated indirectly, using the island model proposed by Crow and Aoki (1984), which corrects the analyses for finite number of populations: 
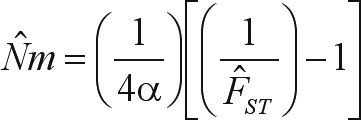
. In this expression, the genetic divergence between populations (*F*_*ST*_) was substituted by θ_*p*_, *R*_*ST*_ and 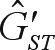, and the correction for a finite number of populations (*n*) was calculated as 
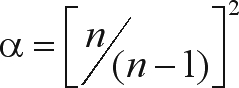
.

###  Mating system analysis

The program MLTR 3.2 (Ritland, 2002), which is based on a mixed outcrossing and correlated outcrossing model, was used to estimate the single and multilocus outcrossing rates for the MS population. We estimated the following population and family parameters: 1) multilocus outcrossing rate (*t*_*m*_) by the maximum likelihood method, 2) single outcrossing rate (*t*_*s*_), 3) the outcrossing rate between related individuals 
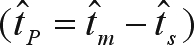
, 4) the paternity correlation (*r*_*p*_) and 5) the selfing correlation (*r*_*s*_) for the population. The standard error of the estimates was obtained through 10,000 bootstraps in which the resampling units were the families.

Null alleles were detected by comparing seed-tree genotypes with their offspring in the MS population using the program MLTR. Null alleles, rather than mistyping, were considered to be present when a homozygous seed-tree mismatched its offspring genotype. In the presence of null alleles the homozygous seed-tree genotype was corrected to a heterozygous form (observed allele/null allele) and the offspring genotypes were corrected through an expected Mendelian segregation ratio of 1:1, as described by Liewlaksaneeyanawin *et al.* (2002).

###  Minimal number of seed-trees

The minimal number of seed-trees 
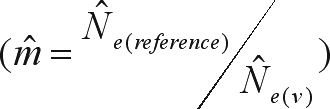
 needed for harvesting seeds in conservation, management and breeding programs was calculated as described by Sebbenn (2002). In this method, 1) *N*_*e(reference)*_ is the minimum effective sample size of seeds to be collected (50, 500 and 1000) and 2) *N*_*e(v)*_ is the variance effective size for a single seed-tree progeny of size *n* (*n* = 100), estimated by



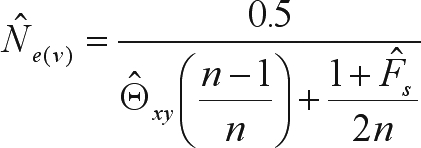


where *F*_*s*_ is the fixation index in offspring and Θ_*xy*_ is the average coancestry coefficient within families, estimated as the half of the relatedness coefficient, 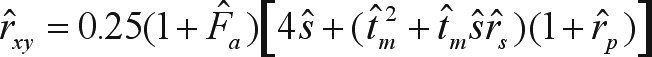, where *F*_*a*_ is the fixation index in the adult population and *s* is the selfing rate, 
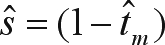
.

###  Paternity analysis

Paternity analysis of each seed was done by maximum-likelihood paternity assignment using the program CERVUS 3.0 (Kalinowski *et al.*, 2007). All 41 of the trees sampled in the MS population were used to determine the putative pollen donor of the seeds. Paternity was based on the Δ statistic (Marshall *et al.*, 1998). The critical value of Δ for each confidence level of paternity analysis was determined by running simulations with CERVUS 3.0 based on 50,000 replications with a 95% confidence level and considering a 0.01 proportion of mistypes. The possibility of selfing was also considered. The total paternity probability of the parent pair was also estimated for all sampled individuals in the population.

###  Spatial genetic structure

Spatial genetic structure (SGS) within the sampled populations was studied using the estimate of the average coancestry coefficient (θ_*xy*_) between all pairs of adult trees based on Loiselle *et al.* (1995). Five consecutive distance classes from 0-196 m (class 1) to 784-980 m (class 5) with 196 m distance intervals were used to plot the SGS correlograms. A 95% confidence interval was calculated for each observed value and each distance class generated from 10,000 permutations of individuals within populations. These analyses were done using the program SPAGEDI version 1.2 (Hardy and Vekemans, 2002).

In the presence of SGS, the fixation index values within populations are over-estimated due to the Wahlund effect (Bittencourt and Sebbenn, 2007). This intrapopulation fixation index (*f*_*i*_) can be corrected by eliminating the Wahlund effect through the application of a relationship for F-statistics described in Bittencourt and Sebbenn (2007) and based on the formula 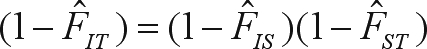 (Wright, 1965). For this correction, Bittencourt and Sebbenn (2007) proposed replacing *F*_*IT*_ and *F*_*ST*_ by *f*_*i*_ and θ_*xyi*_, respectively, such that the corrected intrapopulation fixation index becomes 
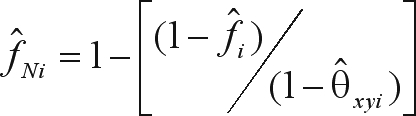
 for the *i*^th^ population.

## Results

###  Genetic diversity

Of the 42 primer pairs tested, nine (21.4%) amplified visible products and were polymorphic in *D. alata* (Table 1). Exclusive and rare alleles were found in all of the populations. Among the nine SSR loci studied, only the MS population showed fixed alleles at locus DO31. The mean loci polymorphism for all populations was 96.3% and the mean number of alleles per population was 3.1 with no significant difference among populations (Table 2). All loci in the three populations where considered independent by linkage disequilibrium analysis (p-value > 0.0013)

The mean observed ( 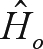) and expected ( 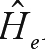) heterozygosities for all loci were 0.342 and 0.619, respectively, and the mean fixation index ( 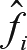) was 0.122 (Table 2). All populations deviated from Hardy-Weinberg equilibrium with significant inbreeding (p-value < 0.0056) (Table 2). A significant total fixation value ( 
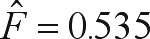
; p-value < 0.0056) was associated with high, significant population differentiation estimates ( 
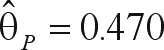
, 
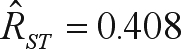
 and 
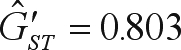
; p-value < 0.0056) and low historic gene flow estimates among populations ( 
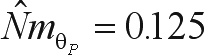
; 
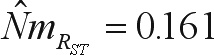
 and 
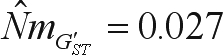
). These findings were indicative of structured populations.

###  Mating system

The estimated outcrossing rate indicated that this species behaved as a mixed-mating system species, with a predominance of outcrossing (Table 3). The multilocus outcrossing rate was significantly less than unity ( 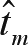 = 0.711; S.E. = 0.061), suggesting selfing and deviation of random mating. The significant difference between the multilocus and single locus outcrossing rates indicated mating among relatives ( 
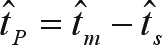
 = 0.135; S.E. = 0.034). The selfing correlation was very high for a tree species ( 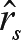 = 0.500; S.E. = 0.076). The paternity estimate correlation was significant and also very high ( 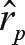 = 0.675; S.E. = 0.084), which led to the conclusion that approximately 48% ( 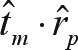) of the offspring derived from correlated matings and were full-sibs, whereas 23% [ 
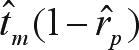
] derived from random matings and were related as half-sibs. The estimated number of effective pollen donors 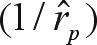 was very low (1.5).

Family outcrossing estimates ( 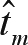) ranged from 0.202 to 0.998 and 19 out of 25 families were significantly different from 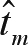 = 1.000 (Table 3). Significant mating rates among relatives were observed in 12 families. Correlated matings ranged from 2.3% to 67.3%, and random matings ranged from 17% to 85.9%. Fourteen families had less than four effective pollen donors.

###  Minimal number of seed-trees

The coancestry coefficient within families ( 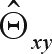 = 0.171) was higher than expected for half-sibs (0.125), consequently the average variance effective size per family ( 
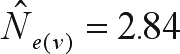
) was lower than expected for this type of family, namely 
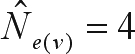
. The fixation index for the seeds was high and significant ( 
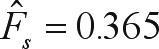
). The minimum number of seed-trees needed for seed harvesting in order to retain the reference effective population sizes of 50, 500 and 1000 was 18, 186 and 352, respectively, with n = 100 seeds per tree.

###  Paternity analysis

The parent pair exclusion probability over eight loci was 0.981. Paternity analysis revealed that of 300 seeds, 132 had a sampled pollen donor within the MS population. Of these 132 seeds, 51 originated by selfing and 81 by outcrossing. The 81 seeds originated by outcrossing had 22 potential pollen donors that dispersed their pollen a mean distance of 610 m (maximum of 1388 m). Dominant pollen donors were present in the 81 seeds originated by outcrossing with seven trees contributing with 62% of the pollen.

###  Spatial genetic structure

**Figure 2:**
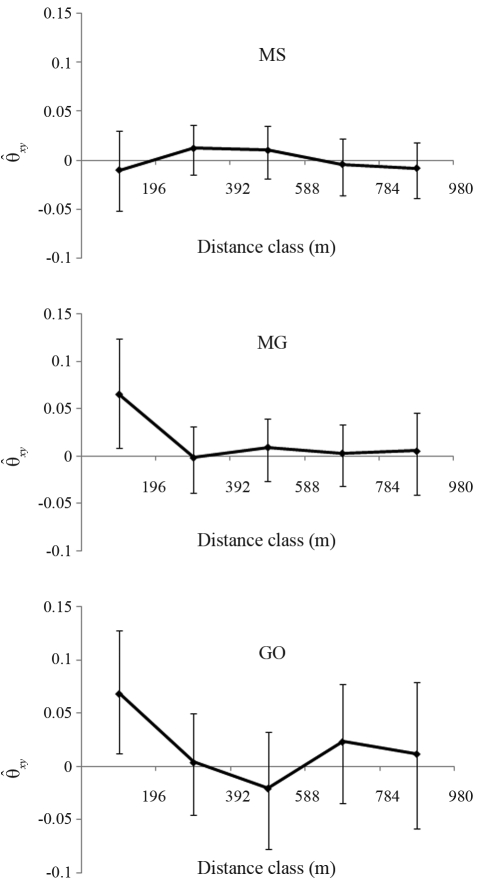
Correlograms for three populations (MS, MG, GO) of *D. alata* showing the average coancestry coefficient (θ_*xy*_) between all pairs of adult trees. The values are the means ± 95% confidence intervals.

Analysis of the spatial genetic structure showed that populations MG and GO had significant coancestry coefficient estimates ( 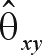 = 0.064 and 0.068, respectively) in the first distance class, indicating SGS up to 196 m (Figure 2). The SGS analysis showed no significant coancestry coefficient estimates for the MS population (Figure 2). Based on the SGS values, the new corrected intrapopulation fixation index values ( 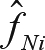) for the MG and GO populations were 0.056 and 0.009, respectively. These findings indicated that the uncorrected 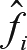 values (MG = 0.117; GO = 0.077) were overestimated by undetected intrapopulation phenomena known as the Wahlund effect.

## Discussion

### *Dipteryx alata* genetic structure and mating system

The genetic diversity values calculated here indicated great genetic diversity in the populations studied. The overall genetic diversity for all of the populations was similar to that of *Dipteryx panamensis* analyzed with nine microsatellite loci (*H*_*e*_ = 0.520-0.604; Hanson *et al.*, 2008).

As the result of a correction that considers the large amount of private alleles among populations, the 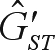 value was nearly twice the value of 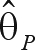 and 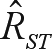 (Hedrick, 2005). The high divergence among the *D. alata* populations in this study indicated population structuring, a fact demonstrated by the existence of correlated matings, selfing and limited seed dispersal. The degree of structure among subpopulations of *D. alata* was very high when compared to that observed in other native tree species of the Brazilian cerrado (Zucchi *et al.*, 2005).

Since we have found evidence of long distance pollen dispersal, we suggest that the main factor structuring the populations is restricted seed dispersal. As SGS is a consequence of different processes, mainly restricted seed dispersal (Dick *et al.*, 2008; Hardy *et al.*, 2006), we suggest that gravity plays a greater role in restricted seed dispersal than the animals, *e.g.*, bats, that usually disperse *D. alata* fruits.

SGS was responsible for subdividing the MG and GO populations into many circular subpopulations with a radius of 196 m that resulted in elevated 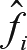 values because of the Wahlund effect. By recognizing SGS within populations, we were able to eliminate the Wahlund effect from the new estimated fixation index values for the MG and GO populations. These findings indicated that besides the possible existence of biparental inbreeding and selfing in the populations, the high 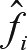 values in the adult populations were also the result of SGS.

The MS population had a high, significant degree of selfing that may impact negatively on heterozygosity and fitness (Lowe *et al.*, 2005). The outcrossing rates determined here were similar to those of congener *D. panamensis* trees located in pasture ( 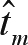 = 0.806-0.865; Hanson *et al.*, 2008), which agrees with the fact that the all seed-trees collected in this study are isolated in pasture. We suggest that isolation and a low plant density may have reduced the MS population outcrossing rates because of the low vector effectiveness in pollination (Steffan-Dewenter and Westphal, 2008), as also observed in other pasture trees (Dick *et al.*, 2003). Our results for the pollen dispersal distances also agreed with those for other insect-pollinated tropical trees that occur in low densities (*e.g.* Dick *et al.*, 2008). The low density of reproductive trees in the studied area resulted in a high number of dominant pollen donors and such dominance has already led to bottlenecks in seeds, thereby reducing their effective size, as demonstrated by the high, significant fixation index( 
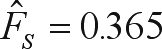
).

###  Implications for genetic conservation and breeding programs

Our data support the existence of high genetic diversity within and among populations of *D. alata*, indicating the possibility of conserving these populations *in situ*. The high divergence among populations revealed that the three studied populations should be treated as evolutionary significant units (ESUs) and management units (MUs) in order to achieve adaptive evolutionary conservation (AEC) objectives in each population (Palsboll *et al.*, 2007). Moreover, *ex situ* conservation strategies and restoration projects should be guided by the levels of genetic diversity and local adaptation so as to avoid problems related to maladaptation (McKay *et al.*, 2005).

Two out of three populations showed significant levels of SGS that generally result in a high fixation index. We suggest that in order to mitigate the effects of SGS when establishing a minimum distance between individuals, future studies should sample all individuals of the same ontogenetic stage in a fixed area; this approach will provide a better understanding of SGS and its influence on genetic structure. The presence of SGS suggests that seed harvesting for genetic conservation and breeding programs needs to be done at a minimum distance of 196 m between seed-trees in order to avoid related seed-parents.

The estimated outcrossing rate suggested the presence of a mixed mating system that is already leading to higher levels of inbreeding. We found evidence of long distance gene flow via pollen that may reduce genetic drift in the small fragmented populations of *D. alata* in the Brazilian cerrado. Nevertheless, the high reproductive dominance indicates that the pollinators are probably not playing their role because of the reduced numbers of reproductive trees and isolation. The low number of effective pollen donors and the expected high number of full-sibs per seed-tree should be taken into account when harvesting seeds for breeding and conservation programs. In this case, to maintain stable population inbreeding levels in *ex situ* conservation programs, at least 18 seed-trees with 100 seeds each are required in order to retain a reference effective size of 50.

## Figures and Tables

**Table 1:** Sequences of the nine primer pairs developed for *D. odorata*, showing the temperature (°C) for primer annealing (*T*_*a*_), sample size (*n*), allele size range (bp), number of alleles per locus (*A*), observed heterozygosity (*H*_*o*_) and expected heterozygosity (*H*_*e*_) of the amplified microsatellite loci in *D. alata*.

Primer^1^	Sequence (F- forward/ R- Reverse)	*T*_*a*_	*n*	bp	*A*	*H*_*o*_	*H*_*e*_
Do 05	F: AgggAggCCAAgAAgTAAgC R: AAggTTTgAAgTTgAAgCTTgg	56	101	198-232	3	0.283	0.543
Do 06	F: AgCggTgAAAAgACCATAgC R: CCAACgATAAgATTCCTCCA	54	101	158-204	6	0.614	0.717
Do 08	F: AgATCAgCggACAAAggTCT R: gTAATgTTgTgCCACTCTTg	58	101	175-205	8	0.377	0.652
Do 17	F: gTTgCTgTCggTTCTCCATA R: CCAAggACgCTgTgCTCTAC	56	101	165-187	3	0.111	0.513
Do 20	F: gCCCATCTAAgCgCATTATT R: AgTggAAgggTggATTgATg	58	101	175-207	5	0.333	0.665
Do 24	F: AACgCAggATCTAgCCAAAA R: CTTCTCgCTgTTgTgCACTC	58	101	183-201	3	0.412	0.508
Do 25	F: AAATgCAAAACggAAgAggA R: CCCCTgAAggAgACTTCgAT	56	101	195-223	4	0.520	0.660
Do 31	F: CAACATgCgATCCTTCCTTT R: gAgAgAAAgAgAgggggTTCA	56	101	198-218	5	0.100	0.497
Do 35	F: CAACCAAAgCAAACAAAgCA R: gCTgAgAAAggggAATgCAg	54	101	208-230	6	0.319	0.649
Average	-	-	101	-	4.780	0.341	0.619

^1^Primers developed by Vinson CC, MSc dissertation, Universidade Federal do Pará, Belém, 2004.

**Table 2:** Estimates of genetic diversity and fixation indices (*f*) for three natural populations of *D. alata* in the cerrado.

Population	*n*	$\hat{P}$	$\hat{A}$	${\hat{H}}_{o}$	${\hat{H}}_{e}$	${\hat{f}}_{i}$
MS	41	89.9	2.9	0.353	0.420	0.161*
MG	30	100	3.1	0.323	0.365	0.117*
GO	30	100	3.2	0.347	0.375	0.077*
Mean over populations	34	96.6	3.1	0.341	0.386	0.134
Mean over loci	101	100	4.7	0.341	0.619	0.122*

*A* = mean number of alleles per locus, *f*_*i*_ = mean fixation index, *H*_*e*_ = mean expected heterozygosity, *H*_*o*_ = mean observed heterozygosity, *n* = sample size, *P* = percent polymorphic loci. *p-value = 0.0056.

**Table 3:** Mating system estimates per family in a *D. alata* population.

Family	${\hat{t}}_{m}$	${\hat{t}}_{P}={\hat{t}}_{m}-{\hat{t}}_{s}$	${\hat{t}}_{m}\cdot {\hat{r}}_{p}$	${\hat{t}}_{m}(1-{\hat{r}}_{p})$	$\left(1/{\hat{r}}_{p}\right)$	*d*_*n*_
1	0.964 ± 0.038	0.118 ± 0.037	0.442	0.522	2.2	1008 (105-1636)
2	0.338 ± 0.123	0.286 ± 0.087	0.029	0.309	11.8	1100 (105-1742)
3	0.398 ± 0.128	-0.391 ± 0.113	0.085	0.313	4.7	846 (131-1315)
4	0.690 ± 0.119	0.045 ± 0.096	0.159	0.531	4.3	856 (131-1246)
5	0.838 ± 0.089	0.139 ± 0.073	0.330	0.508	2.5	824 (238-1458)
6	0.679 ± 0.126	-0.203 ± 0.112	0.105	0.574	6.5	707 (238-1352)
7	0.355 ± 0.119	-0.250 ± 0.092	0.055	0.300	6.5	643 (65-1199)
8	0.431 ± 0.128	-0.228 ± 0.094	0.058	0.373	7.5	731 (174-1373)
9	0.918 ± 0.069	0.115 ± 0.058	0.218	0.700	4.2	603 (65-1200)
10	0.709 ± 0.116	-0.171 ± 0.106	0.398	0.311	1.8	543 (129-1207)
11	0.998 ± 0.005	0.031 ± 0.003	0.408	0.590	2.4	485 (80-1125)
12	0.962 ± 0.041	0.102 ± 0.038	0.103	0.859	9.3	633 (274-1464)
13	0.202 ± 0.101	-0.495 ± 0.085	0.032	0.170	6.4	518 (86-1246)
14	0.862 ± 0.089	0.016 ± 0.079	0.555	0.307	1.6	546 (102-1351)
15	0.840 ± 0.087	0.211 ± 0.064	0.617	0.223	1.4	653 (58-1522)
16	0.998 ± 0.005	0.050 ± 0.006	0.319	0.679	3.1	549 (86-1326)
17	0.886 ± 0.077	0.050 ± 0.069	0.480	0.406	1.8	855 (70-1742)
18	0.918 ± 0.067	0.197 ± 0.058	0.400	0.518	2.3	824 (80-1733)
19	0.958 ± 0.039	0.093 ± 0.036	0.487	0.471	2.0	733 (113-1629)
20	0.404 ± 0.128	-0.344 ± 0.100	0.075	0.329	5.4	585 (75-1200)
21	0.923 ± 0.063	0.133 ± 0.058	0.673	0.250	1.4	521 (41-1993)
22	0.998 ± 0.005	0.064 ± 0.008	0.587	0.411	1.7	513 (77-1043)
23	0.763 ± 0.105	0.020 ± 0.083	0.310	0.453	2.5	487 (90-1010)
24	0.546 ± 0.129	-0.248 ± 0.106	0.093	0.453	5.9	465 (63-978)
25	0.998 ± 0.005	0.158 ± 0.018	0.541	0.457	1.8	487 (56-906)
Population	0.711 ± 0.061	0.135 ± 0.034	0.480	0.230	1.5	-

*d*_*n*_ = mean distance to the nearest neighbor and ( ) range in meters, *t*_*m*_ = multilocus outcrossing rate, ${\hat{t}}_{P}={\hat{t}}_{m}-{\hat{t}}_{s}$ = outcrossing rate between related individuals, ${\hat{t}}_{m}\cdot {\hat{r}}_{p}$ = correlated matings, ${\hat{t}}_{m}(1-{\hat{r}}_{p})$ = random matings and $\left(1/{\hat{r}}_{p}\right)$ = effective pollen donors.
